# Bipartite graph-based collaborative matrix factorization method for predicting miRNA-disease associations

**DOI:** 10.1186/s12859-021-04486-w

**Published:** 2021-11-27

**Authors:** Feng Zhou, Meng-Meng Yin, Cui-Na Jiao, Zhen Cui, Jing-Xiu Zhao, Jin-Xing Liu

**Affiliations:** The School of Computer Science, Qufu Normal University, Rizhao, 276826 China

**Keywords:** MiRNA–disease associations association prediction, Matrix factorization, Bipartite graph, Gaussian interaction profile

## Abstract

**Background:**

With the rapid development of various advanced biotechnologies, researchers in related fields have realized that microRNAs (miRNAs) play critical roles in many serious human diseases. However, experimental identification of new miRNA–disease associations (MDAs) is expensive and time-consuming. Practitioners have shown growing interest in methods for predicting potential MDAs. In recent years, an increasing number of computational methods for predicting novel MDAs have been developed, making a huge contribution to the research of human diseases and saving considerable time. In this paper, we proposed an efficient computational method, named bipartite graph-based collaborative matrix factorization (BGCMF), which is highly advantageous for predicting novel MDAs.

**Results:**

By combining two improved recommendation methods, a new model for predicting MDAs is generated. Based on the idea that some new miRNAs and diseases do not have any associations, we adopt the bipartite graph based on the collaborative matrix factorization method to complete the prediction. The BGCMF achieves a desirable result, with AUC of up to 0.9514 ± (0.0007) in the five-fold cross-validation experiments.

**Conclusions:**

Five-fold cross-validation is used to evaluate the capabilities of our method. Simulation experiments are implemented to predict new MDAs. More importantly, the AUC value of our method is higher than those of some state-of-the-art methods. Finally, many associations between new miRNAs and new diseases are successfully predicted by performing simulation experiments, indicating that BGCMF is a useful method to predict more potential miRNAs with roles in various diseases.

## Background

MicroRNAs (miRNAs) are single-stranded small ncRNAs with a typical length of 19 ~ 25 nt [1]. Although they do not encode proteins, they play a significant role in regulating gene expression. They usually silence gene expression through translational repression or otherwise function as post-transcriptional gene regulators. In 1993, the first miRNA, lin-4, was discovered by Victor Ambros et al*.* [2]. After seven years, biological researchers discovered the second miRNA, let-7 [3]. As miRNAs are increasingly identified as playing crucial roles, researchers have begun to focus more attention on identifying miRNAs.

Studies have found that miRNAs are crucial components in cells and can play roles in many important biological processes, including haematopoiesis, cell proliferation, development, differentiation, apoptosis, cell ageing, viral infection, embryonic development and organ formation [4–7]. Mutated and disordered miRNAs will lose the ability to control their target genes, leading to the development of various complex human diseases, such as cardiovascular diseases, nervous system diseases, tumours, metabolic diseases, and autoimmune diseases [8, 9]. As an example, a miR-133b defect is easily observed in the midbrain of patients with Parkinson's disease; miR-133b is thought to have a regulatory effect on the maturation and function of midbrain dopamine neurons [10]. In addition, Gao et al. found that the expression of miR-155 in the serum of lung cancer patients was much higher than that in normal samples by experimental PCR [11]. Furthermore, Takamizawa et al. have proved that the homology of let-7 is significantly reduced in the process of lung cancer [12]. However, discovering meaningful associations between miRNAs and diseases is a time-consuming process. Therefore, it is urgent to develop fast and efficient computational methods for predicting miRNA–disease associations.

In the last decade, a large number of methods and models have been proposed to identify potential relationships between miRNAs and diseases [13, 14]. These methods and models have mainly focused on solving the above problem by machine learning, network mining, combinatorial optimization, and related approaches [15–17]. For example, Jiang et al. used a support vector machine to extract data on positive samples from negative samples. This method extracted features from miRNA target data and phenotypic similarity data and achieved favourable results [18]. Chen et al. applied the random walk algorithm with a restart, which is also a classic network-based prediction model, to miRNA–disease association (MDA) prediction [19]. In 2013, Qabaja et al. proposed a protein–protein interaction network based on the lasso regression model. They first used the lasso regression model to identify miRNAs associated with markers of diseases and then integrated biological networks and multisource data to define the gene signatures of miRNAs and diseases. Finally, this method achieved good predictive performance [20]. Xuan et al. proposed a prediction method based on the K-nearest neighbour algorithm. This method constructs a similarity network by integrating the miRNA–disease phenotype similarity network, the family information of miRNAs, and the relationships between diseases and miRNAs identified by biological experiments. The disadvantage of this method is that it cannot be applied to the association prediction of diseases without any known related miRNAs [21].

In 2014, Chen et al. proposed a semi-supervised algorithm named regularized least squares (RLSMDA) to predict potential disease-miRNA associations. The advantage of this method is that it does not require negative MDAs information and can be applied to the prediction of isolated diseases. In 2017, Chen et al. proposed a predictive model for the associations between miRNAs and diseases based on Laplacian regularized sparse subspace learning (LRSSLMDA) [22]. They used Laplacian regularization to keep local information and then used the $L_{1}$ norm to select important miRNA/disease features, further improving the precision of the algorithm. Chen et al. proposed a computational method, ensemble learning and link prediction for miRNA-disease association (ELLPMDA), which combines both machine learning methods and similarity-based algorithms. This method is based on a globally similar measurement method for diseases without any associated miRNAs [23]. Algorithms such as neural networks are also used to predict miRNA–disease associations. In 2017, Fu et al. proposed a deep integration model (DeepMDA), which uses a stack-type autoencoder to extract high-level features from similar information and then predicts disease-miRNA associations through a three-layer neural network [24]. In addition, matrix factorization is also used to predict the association between miRNAs and diseases. In 2019, Gao et al. proposed a computational model, dual-network sparse graph regularized matrix factorization (DNSGRMF), for predicting miRNA–disease associations by integrating the miRNA functional similarity matrix, the disease semantic similarity matrix and Gaussian kernel similarities with the addition of the $L_{2,1}$ norm. They used collaborative matrix factorization to predict miRNA–disease associations [25]. Later, a more efficient miRNA-disease associations prediction model, nearest profile-based collaborative matrix factorization (NPCMF), was proposed by Gao et al., which integrates Gaussian kernel similarity and the nearest profile, taking the nearest neighbour information into account. Finally, DNSGRMF and NPCMF achieved excellent predictive accuracy based on fivefold cross-validation [26].

Although there are many advanced methods to predict MDAs, they still have some shortcomings. For instance, several methods trigger bias to miRNAs (diseases). Moreover, the small number of known relationships cannot be utilized to predict new miRNAs and diseases. More importantly, in some methods, the nearest neighbour information of the miRNA and the disease is not considered. To address the limitations of previous methods, a computational method of bipartite graphs based on collaborative matrix factorization (BGCMF) is proposed. The specific contributions of our method include the following two aspects:

- In our method, the miRNA similarity matrix and disease similarity matrix are constructed by combining Gaussian interaction kernel similarity, miRNA functional similarity, and disease semantic similarity. This could help to infer potential miRNA–disease associations.
- It is worth noting that the bipartite graph algorithm (BG) is introduced to our method for maximum consideration of neighbouring information for miRNAs and diseases. Then, it calculates a weighted average of similarities between miRNAs and diseases to eliminate the bias on prediction.

In addition, there are quite a few missing associations in the original matrix ${\mathbf{Y}}$, and Weight K Nearest Known Neighbours (WKNKN) [27] is implemented as a pre-treatment step to minimize the error. Moreover, five-fold cross-validation is exploited in our method to evaluate our experimental results. We also introduce simulation experiments to further evaluate the performance of our method. Overall, the results demonstrate that our BGCMF method is superior to other existing advanced methods.

## Results

### Human miRNA–disease associations association dataset

In this study, the gold standard human miRNA–disease association dataset was downloaded from the Human microRNA Disease Database (HMDD) v2.0 [28]. HMDD v2.0 includes 495 miRNAs, 383 diseases and 5430 experimentally verified miRNA-disease associations. In this paper, the dataset includes three matrices: the adjacency matrix ${\mathbf{Y}}$, the miRNA functional similarity matrix ${\mathbf{S}}_{m}$, and the disease semantic similarity matrix ${\mathbf{S}}_{d}$. The information for the dataset is listed in Table 1.

**Table 1:** The gold standard dataset of miRNAs, diseases and associations

Dataset	Gold standard dataset
MiRNAs	495
Diseases	383
Associations	5430

We use an adjacency matrix ${\mathbf{Y}} \in {\mathbb{R}}^{n \times m}$ to describe the associations between miRNAs and diseases that have been validated, where $n$ represents the number of miRNAs and $m$ represents the number of diseases. When $M\left( i \right)$ and $D\left( j \right)$ are associated, ${\mathbf{Y}}\left( {M\left( i \right),D\left( j \right)} \right)$ is set to 1; otherwise, ${\mathbf{Y}}\left( {M\left( i \right),D\left( j \right)} \right)$ is set to 0. The following is the expression of the matrix ${\mathbf{Y}}$:

1 $${\mathbf{Y}}\left( {M\left( i \right),D\left( j \right)} \right) = \left\{ {\begin{array}{*{20}c} {1,} & {M\left( i \right){\text{ is associated with disease }}D\left( j \right),} \\ {0,} & {{\text{otherwise}}{.}} \\ \end{array} } \right.$$

### Performance evaluation

In this study, we implement fivefold cross-validation to evaluate the prediction performance of each method. The principle of fivefold cross-validation is to randomly divide the known miRNA-disease associations into five subsets, one of which is used as a test set, and the rest are used as a training set. Then, five models are trained by cycling five times, and the average of the five evaluation results is calculated as the final score of the model. Finally, fivefold cross-validation was performed 100 times, and the final score was taken as the average. It is worth noting that in our BGCMF method, WKNKN is used as a preprocessing procedure to evaluate unknown MDAs. At the same time, the nearest neighbour information is applied to our method, and it has the advantage of taking into account the nearest neighbour information and improving the accuracy of the prediction.

To verify the effect of the prediction, the area under the curve (AUC) value was applied in this study, which is widely used in previous studies. Therefore, a receiver operating characteristic (ROC) curve was obtained. In this curve, the x-axis is the false-positive rate (FPR, specificity), and the y-axis is the true positive rate (TPR, sensitivity). The definitions for calculating specificity and sensitivity are as follows:

2 $$Specificity = \frac{TN}{{TN + FP}},$$

3 $$Sensitibity = \frac{TP}{{TP + FN}},$$

where $TP$ represents the number of positive samples, $FP$ represents the number of false-positive samples, $TN$ represents the number of negative samples and $FN$ represents the number of false-negative samples. An AUC value between 0.5 and 1 is considered feasible, with $AUC = 1$ representing the best predictive performance and $AUC = 0.5$ representing stochastic prediction.

### Comparison with other methods

**Fig. 1:**
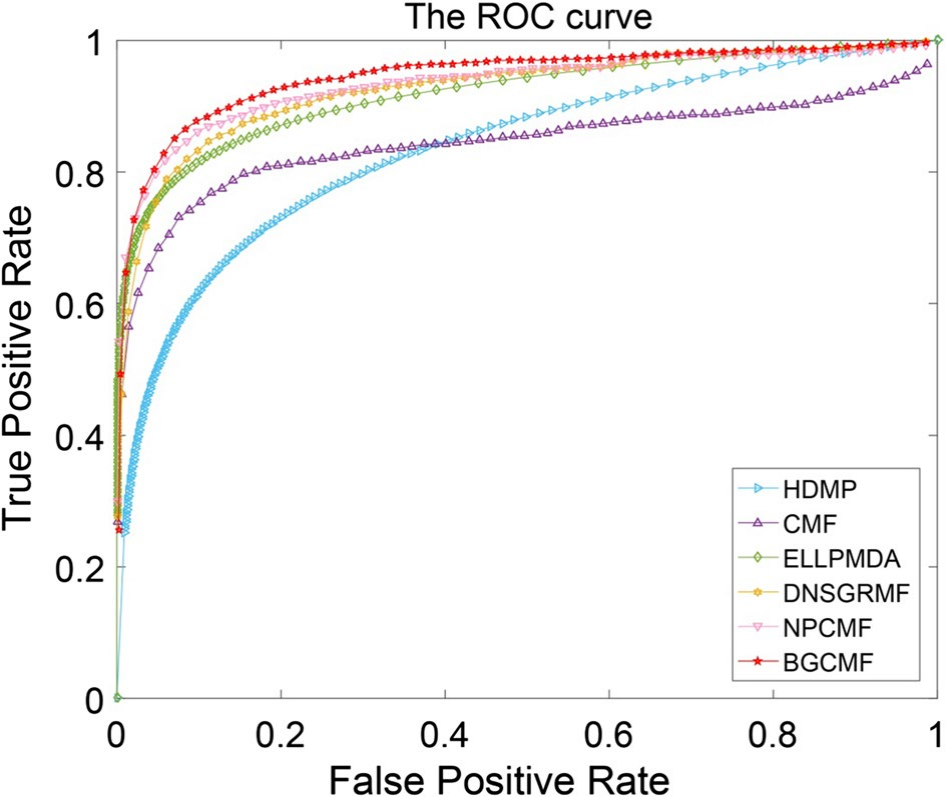
The ROC curve for HDMP, CMF, ELLPMDA, DNSGRMF, NPCMF and BGCMF in fivefold cross validation experiment, respectively

Based on experimentally confirmed associations between diseases and miRNAs, fivefold cross-validation is implemented in this paper to evaluate the predictive accuracy of BGCMF. We compared our method with other advanced methods, such as HDMP [21], CMF [29], ELLPMDA [23], DNSGRMF [25] and NPCMF [26]. The experimental results are listed in Table 2. More intuitively, the ROC curves are shown in Fig. 1. The AUCs of HDMP, CMF, ELLPMDA, DNSGRMF and NPCMF were 0.8342, 0.8697, 0.9193, 0.9304, and 0.9429, respectively, while the AUC of BGCMF was 0.9514. The best value is in bold.

**Table 2:** The AUC results of fivefold cross validation experiments

Methods	AUC
HDMP	0.8342(0.0010)
CMF	0.8697(0.0011)
ELLPMDA	0.9193(0.0002)
DNSGRMF	0.9304(0.0011)
NPCMF	0.9429(0.0011)
**BGCMF**	**0.9514(0.0007)**

From the above statistical results, our method is 11.72% higher than the lowest value of HDMP. The BGCMF is 2.1% and 0.85% higher than the values of DNSGRMF and NPCMF, respectively. Therefore, we can conclude from the experimental results that the BGCMF has excellent predictive performance.

### Sensitivity analysis from WKNKN

**Fig. 2:**
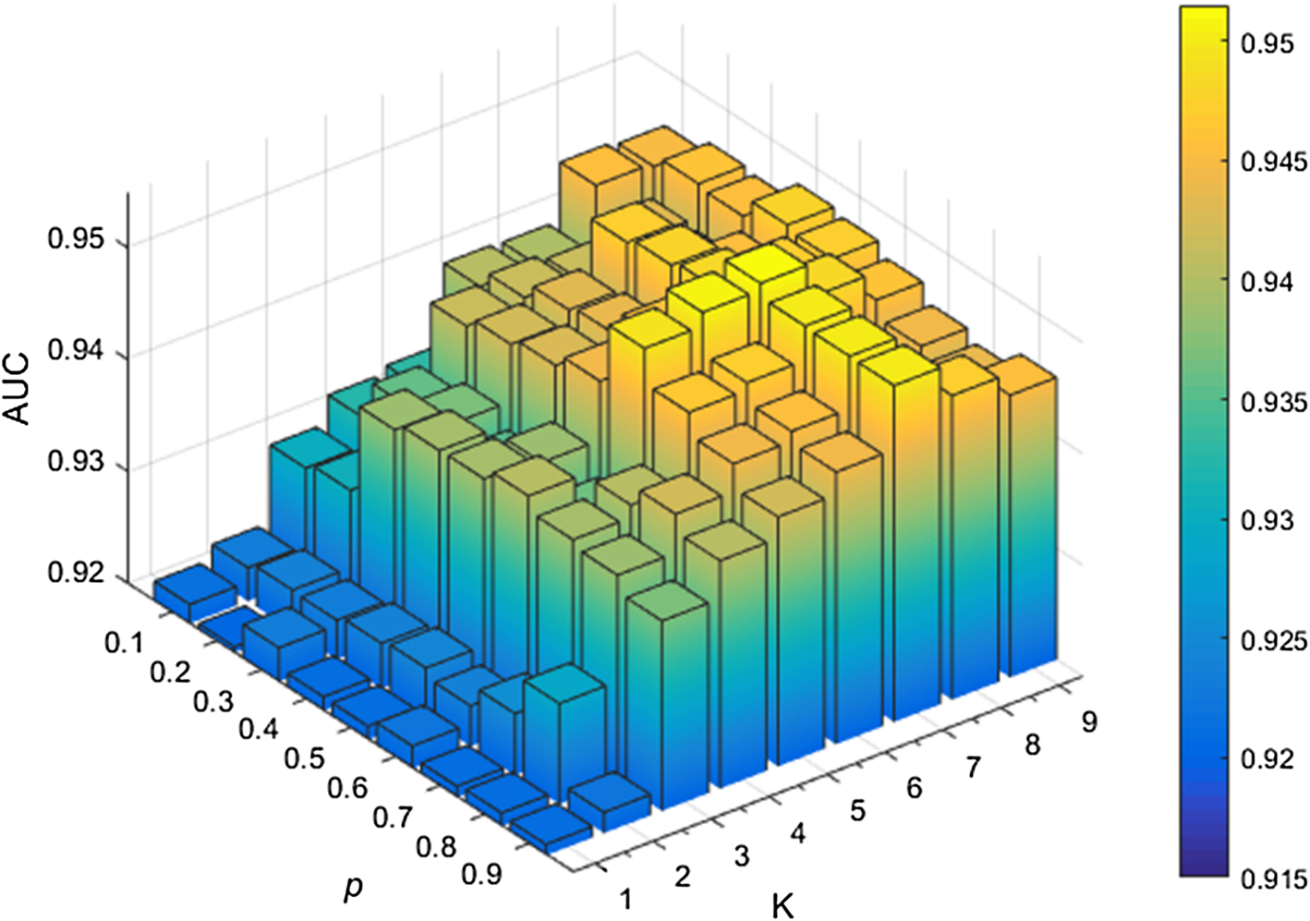
Sensitivity analysis from WKNKN

If a miRNA or a disease is known, it must have one or more associations. However, there are many missing unknown associations in the interaction matrix ${\mathbf{Y}}$. WKNKN pre-processing is used to estimate the interaction possibilities to minimize the error. There are two parameters $K$ and $p$ in WKNKN, where $K$ represents the number of known nearest neighbours and $p$ represents the decay term for the neighbour. The value of $p$ is between 0 and 1. As shown in Fig. 2, when $K = 7$ and $p = 0.6$, the AUC value tends to be stable.

### Case study

The previous sections verify that our proposed method has outstanding predictive performance. Colon, prostate, and kidney are selected in the case study to further illustrate the superior performance of our BGCMF. The known miRNA–disease associations in the standard dataset are used as a training set to predict potential disease-associated miRNAs. Our specific process first uses the BGCMF method to predict these three diseases, and the choice of parameters is as described above. Then, the predicted score matrix is compared to the original miRNA-disease association matrix. The associations of predicted scores with changes are filtered and compared. Finally, we validated whether the predicted new miRNA–disease associations exist in the updated dbDEMC [30], miR2Disease [31] and the HMDD v3.2 [32].

Colon neoplasms, also known as bowel cancer, are one of the three most common cancers, accounting for 10% of all cancer cases. Due to the low detection rate of colon tumours in the early stages, it creates a huge threat to people’s lives. New biomarkers may help to improve the early detection of colon tumours. Recent studies have found that miRNA dysregulation can be used as a marker for colon tumour diagnosis in colon neoplasm cells. For example, miR-145 and miR-126 can inhibit the growth of colon neoplasm cells, and an increasing number of miRNAs associated with colon neoplasms have been found to be of great significance for improving the early detection of colon neoplasms. Here, the first type of case is colon neoplasms. In the dataset used in our experiments, there are 5 existing associations between miRNAs and colon neoplasms. After the simulation experiment is performed, the top 30 miRNAs of colon neoplasms are extracted, and all existing associations are successfully predicted. At the same time, 25 novel MDAs are predicted. Among these 25 new miRNAs, all of the miRNAs are validated by dbDEMC, miR2Disease and HMDD v3.2. More importantly, fourteen of them are confirmed by the above three databases. For example, in 2007, Shi et al. found that the target gene of miR-145 is insulin receptor substrate-1 and can inhibit the growth of colon cancer cells [33]. In 2013, Wan et al*.* identified that patients with colon cancer with high expression of miR-199a-3p had a lower survival rate [34]. Table 3 lists the simulation results of colon tumours, and the known associations are shown in bold. *I, II, III* represent dbDEMC, miR2Disease and the HMDD v3.2.

**Table 3:** Prediction MIRNAs for colon neoplasms

Rank	miRNA	Evidence	Rank	miRNA	Evidence
1	**hsa-mir-145**	**Known**	16	hsa-mir-19b	*I;II*
2	**hsa-mir-1**	**Known**	17	hsa-let-7a	*I; II; III*
3	**hsa-mir-106a**	**Known**	18	hsa-mir-200b	*I; III*
4	**hsa-mir-126**	**Known**	19	hsa-mir-34a	*I; II; III*
5	**hsa-mir-17**	**Known**	20	hsa-mir-221	*I; II; III*
6	hsa-mir-143	*I; II; III*	21	hsa-mir-200c	*I; II; III*
7	hsa-mir-21	*I; II; III*	22	hsa-mir-146a	*I; III*
8	hsa-mir-155	*I; II; III*	23	hsa-mir-141	*I; II; III*
9	hsa-mir-20a	*I; II; III*	24	hsa-mir-19a	*I; II; III*
10	hsa-mir-125b	*I; III*	25	hsa-mir-29a	*I; II; III*
11	hsa-mir-9	*I; II*	26	hsa-mir-200a	*III*
12	hsa-mir-22	*I; III*	27	hsa-mir-142	*III*
13	hsa-mir-16	*I; III*	28	hsa-mir-7	*I*
14	hsa-mir-31	*I; II; III*	29	hsa-mir-92a	*III*
15	hsa-mir-18a	*I; II; III*	30	hsa-let-7b	*I; II; III*

The next case is prostate neoplasms, which are the third most common cause of male cancer-related death. In our simulation experiments, we also select the top 30 miRNAs with the highest correlation scores, and seven known miRNAs associated with prostate neoplasms are successfully predicted. Among the 23 newly predicted miRNAs associated with prostate neoplasms, miR143, miR21, and miR126 are the highest ranked miRNAs, as confirmed by three databases at the same time. Only miR-200b is not confirmed in either dbDEMCs or miR2Disease associated with prostate neoplasms, but it is confirmed by HMDD v3.2. Although a large number of miRNAs have been discovered, knowledge regarding their function and physiological/pathological significance remains limited. Table 4 lists the details of the experiment and the existing associations.

**Table 4:** Prediction MIRNAs for prostate neoplasms

Rank	miRNA	Evidence	Rank	miRNA	Evidence
**1**	**hsa-mir-125b**	**Known**	16	hsa-mir-100	*I; III*
**2**	**hsa-mir-1**	**Known**	17	hsa-mir-375	*I; III*
**3**	**hsa-mir-183**	**Known**	18	hsa-mir-20a	*I; III*
**4**	**hsa-mir-145**	**Known**	19	hsa-mir-31	*I; III*
**5**	**hsa-mir-99a**	**Known**	20	hsa-mir-7	*I; III*
**6**	**hsa-mir-9**	**Known**	21	hsa-mir-96	*I; III*
**7**	**hsa-mir-574**	**Known**	22	hsa-mir-200a	*I; III*
8	hsa-mir-143	*I; II; III*	23	hsa-mir-200b	*III*
9	hsa-mir-21	*I; II; III*	24	hsa-mir-34a	*I; II; III*
10	hsa-mir-126	*I; II; III*	25	hsa-mir-141	*I; III*
11	hsa-mir-182	*I; II; III*	26	hsa-mir-221	*I; III*
12	hsa-mir-133a	*I; III*	27	hsa-mir-155	*I; III*
13	hsa-mir-199a	*I; II; III*	28	hsa-mir-17	*II; III*
14	hsa-mir-223	*I; II; III*	29	hsa-mir-200c	*I; III*
15	hsa-mir-22	*I; III*	30	hsa-mir-146a	*II; III*

**Fig. 3:**
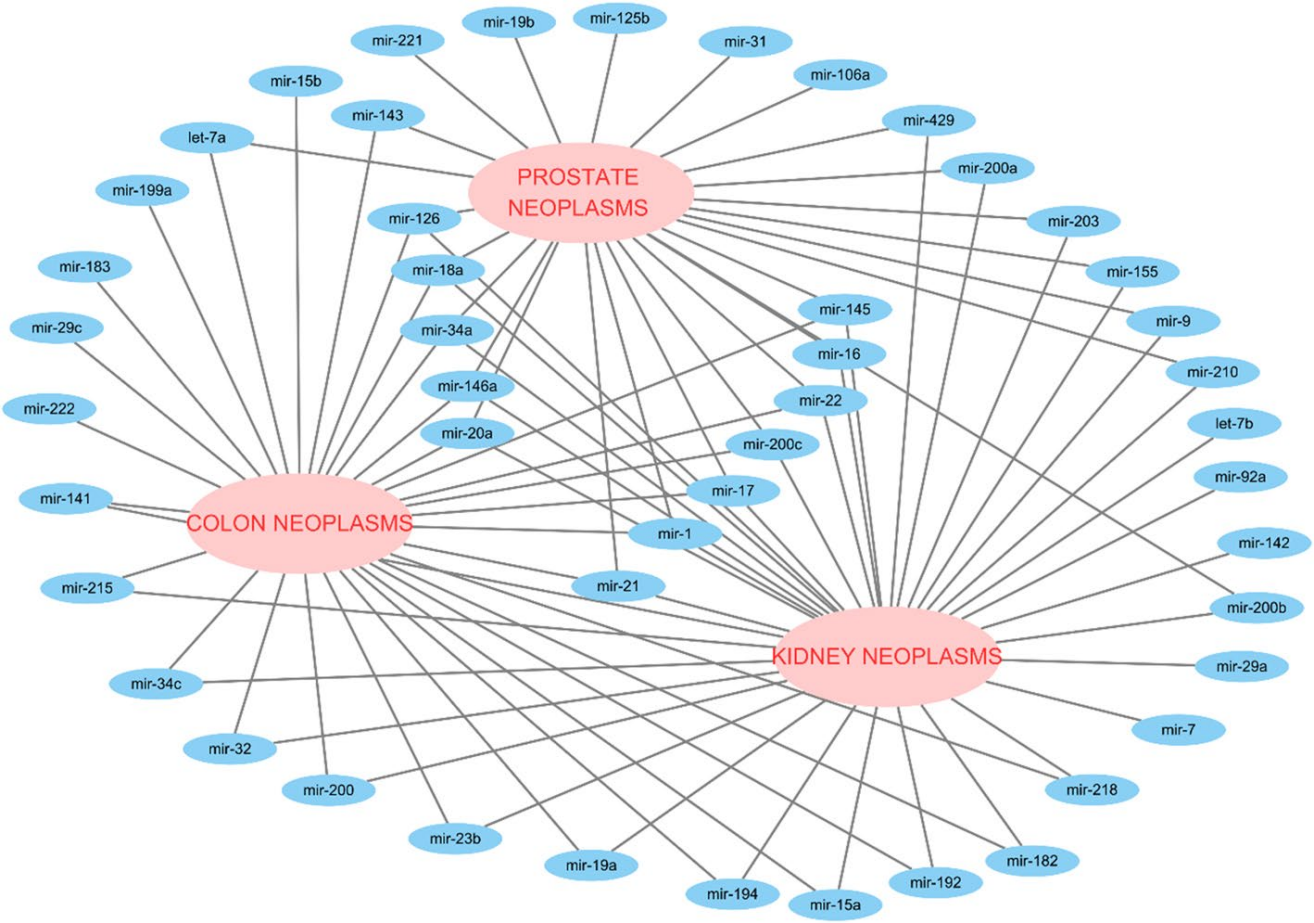
Visualized miRNA–disease associations association network of case study

The last case is kidney neoplasms. Kidney neoplasms, also known as kidney cancer, are cancers that originate in kidney cells and include several different types of tumours. Kidney neoplasms account for 3% of adult malignancies [35]. According to previous studies, a large number of miRNAs have been examined for kidney tumours. For example, circulating levels of miR-15b in patients with advanced kidney cancer are significantly reduced [36]. In addition, overexpression of miR-210 leads to the amplification of renal cancer cell centrosomes [37]. In this case, there are 9 miRNAs that have associations with kidney neoplasms. Nine known miRNAs are successfully predicted in our results. Simultaneously, 35 new miRNAs are predicted. Among the 35 new miRNAs, 29 miRNAs connected with kidney neoplasms have discovered experimental proof from three databases. For example, studies have found that miR-17 is carcinogenic and overexpressed in renal cell carcinoma. Although the predicted novel miRNAs, including miR205, miR125b, miR-7, miR-221, miR-31, and miR-92a, are unconfirmed by miR2Disease or dbDEMC, these miRNAs are closely associated with kidney neoplasms. Table 5 lists the simulation results of kidney neoplasms, and the known associations are shown in bold. To show the simulation experiment of BGCMF more intuitively, Cytoscape software was used to map the three predicted disease-miRNA association networks. As shown in Fig. 3, large ellipses indicate the three diseases, and small ellipses indicate the predicted miRNAs.

**Table 5:** Prediction MIRNAs for kidney neoplasms

Rank	miRNA	Evidence	Rank	miRNA	Evidence
1	**hsa-mir-141**	**Known**	23	hsa-mir-34a	*I*
2	**hsa-mir-15a**	**Known**	24	hsa-mir-126	*I; II; III*
3	**hsa-mir-21**	**Known**	25	hsa-mir-146a	*I*
4	**hsa-mir-1**	**Known**	26	hsa-mir-7	unconfirmed
5	**hsa-mir-192**	**Known**	27	hsa-mir-221	unconfirmed
6	**hsa-mir-200c**	**Known**	28	hsa-mir-17	*II; III*
7	**hsa-mir-215**	**Known**	29	hsa-mir-31	unconfirmed
8	**hsa-mir-23b**	**Known**	30	hsa-mir-92a	unconfirmed
9	**hsa-mir-200**	**Known**	31	hsa-mir-15b	*I*
10	hsa-mir-200b	*I; II; III*	32	hsa-mir-19a	*I*
11	hsa-mir-200a	*I; III*	33	hsa-mir-143	*I*
12	hsa-mir-9	*I*	34	hsa-mir-29c	*I; III*
13	hsa-mir-16	*I*	35	hsa-mir-183	*I; III*
14	hsa-mir-155	*I; III*	36	hsa-let-7a	*I*
15	hsa-mir-210	*I; II*	37	hsa-mir-222	*I*
16	hsa-mir-429	*I*	38	hsa-mir-199a	*I; II; III*
17	hsa-mir-203	*I*	39	hsa-mir-182	*I; II*
18	hsa-mir-205	Unconfirmed	40	hsa-mir-32	*I*
19	hsa-mir-125b	Unconfirmed	41	hsa-mir-18a	*I*
20	hsa-mir-20a	*I; II*	42	hsa-mir-194	*I*
21	hsa-mir-145	*I*	43	hsa-mir-34c	*I*
22	hsa-mir-22	*I*	44	hsa-mir-218	*I*

## Discussion

**Fig. 4:**
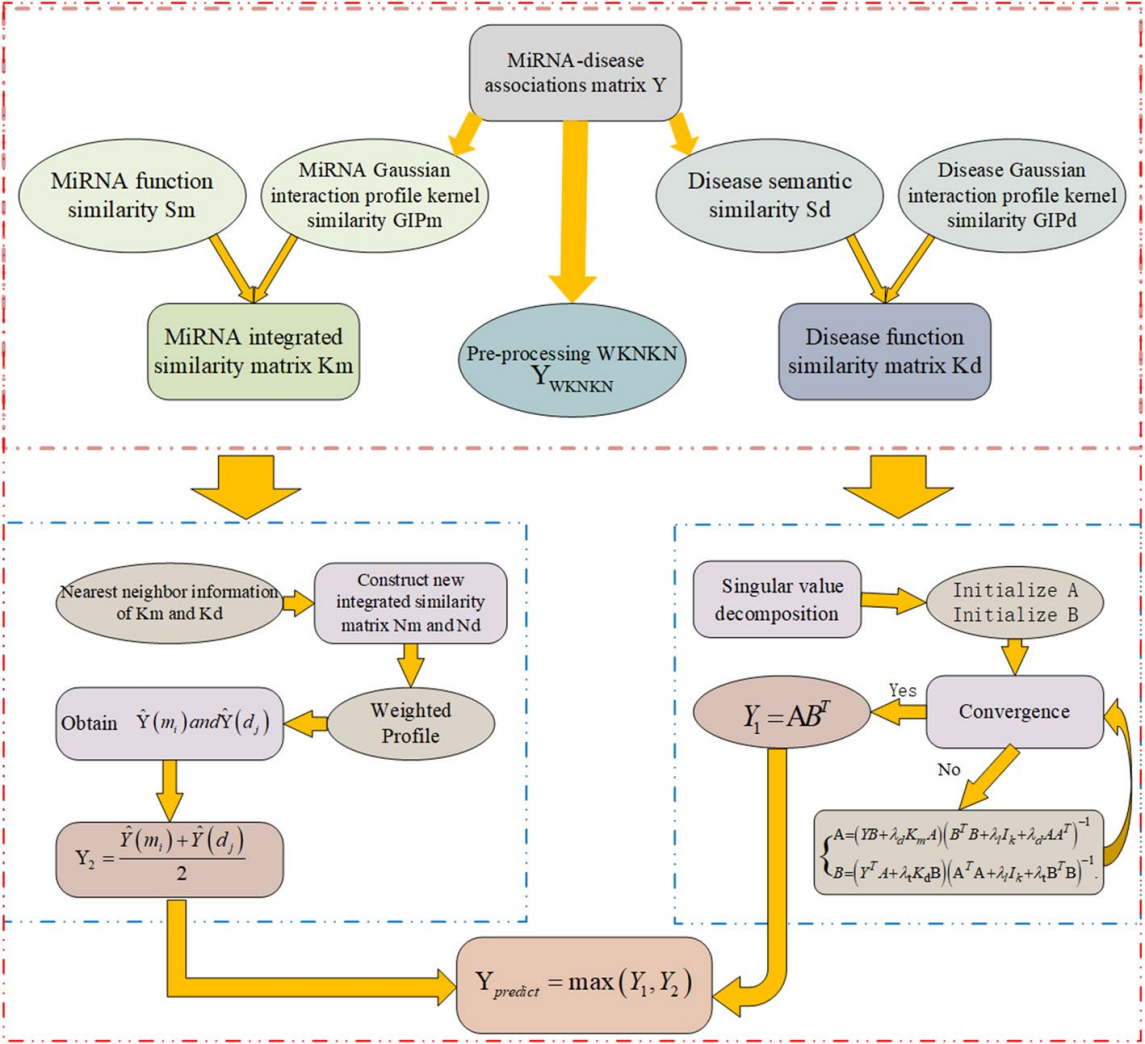
Flow chart of BGCMF in novel MDAs prediction by integrating miRNA function similarity, disease semantic similarity, and known miRNA-disease associations. Then combine the prediction results of the BG algorithm and CMF algorithm to obtain the final prediction matrix

MiRNAs are involved in many physiological processes, such as organismal development, cell differentiation and proliferation, apoptosis, hormone secretion, and lipid metabolism. miRNAs are closely related to the occurrence and development of tumours, metabolic diseases, stress diseases, and cardiovascular diseases. With the development of miRNA bioinformatics, direction prediction and other advances in biological science and technology, a large number of miRNAs have been discovered and verified. However, validating the associations between miRNAs and diseases through biological experiments is time-consuming and expensive. Therefore, it is absolutely necessary to develop new and effective computational models to predict potential associations between miRNAs and diseases. In this paper, an efficient and useful method to predict potential MDAs is developed. Our method can be divided into three parts. The entire calculation process is described in detail in Fig. 4. The first step in this method is to process the data for subsequent prediction. Then, we use the CMF algorithm and BG algorithm to make predictions based on the processed data separately. Finally, we combine the prediction results of the two algorithms to obtain the final prediction matrix. The BGCMF achieves an overall result that is better than the two results given by single models.

## Conclusions

The success of our method can be mainly attributed to several factors. First, we combined Gaussian interaction profile similarity with miRNA functional similarity and disease semantic similarity to obtain accurate information about miRNA pairs and disease pairs. In addition, WKNKN is an essential pretreatment process. It is worth noting that our largest contribution is combining the bipartite graph algorithm with the collaborative matrix factorization model. This allows for maximum consideration of neighbouring information for miRNAs and diseases, preventing the network similarity of miRNAs and diseases from being affected. Finally, both fivefold cross-validation results and three kinds of case studies on colon neoplasms, prostate neoplasms, and kidney neoplasms demonstrated the reliable prediction performance of BGCMF.

In the future, an increasing number of useful methods will be applied to predict potential MDAs. We will continue to study this aspect of research. At the same time, more meaningful datasets are being published in online bio-databases. Therefore, our next work will focus on developing effective methods to predict novel miRNA–disease associations and to evaluate the effectiveness of the method on diverse datasets.

## Method

This novel method is named bipartite graph-based collaborative matrix factorization (BGCMF). The method is divided into two major steps. First, the Gaussian interaction profile kernel (GIP) and nearest neighbour profile (NP) are introduced in our method to process the original miRNA matrix and the disease matrix to obtain their network information. At the same time, WKNKN is used to handle the original interaction matrix ${\mathbf{Y}}$ to minimize the error. Second, the BG algorithm is implemented to obtain prediction matrix ${\mathbf{Y}}_{{1}}$ and collaborative matrix factorization (CMF) to obtain the prediction matrix ${\mathbf{Y}}_{2}$, respectively. Finally, the prediction matrix ${\mathbf{Y}}_{{{\text{predict}}}}$ is obtained by combining our two improved models. The flowchart of BGBMF is shown in Fig. 4.

### MiRNA functional similarity

With the hypothesis that functionally similar miRNAs tend to be associated with phenotypically similar diseases, a computing method of miRNA functional similarity was presented by Wang et al*.* [10]. The functional similarity score matrix can be downloaded from http://www.cuilab.cn/files/images/cuilab/misim.zip. Here, the obtained functional similarity for miRNA is denoted by ${\mathbf{S}}_{m} \in {\mathbb{R}}^{{{\text{n}} \times {\text{n}}}}$, and the value of entity ${\mathbf{S}}\left( {M\left( i \right),M\left( j \right)} \right)$ measures the closeness between miRNA $M\left( i \right)$ and $M\left( j \right)$.

### Disease semantic similarity

A directed acyclic graph (DAG) is proposed to describe the relationships among various diseases. In addition, the disease $D$ can be described by $DAG\left( D \right) = \left( {D,T\left( D \right),E\left( D \right)} \right)$. $T\left( D \right)$ is the node set and represents both its ancestor nodes and $D$ itself. $E\left( D \right)$ is used to represent all direct edges between child nodes and parent nodes. The semantic similarity value of disease $D$ is as follows:

4 $$SV1\left( {\text{D}} \right) = \sum\limits_{d \in T\left( D \right)} {D1_{D} } \left( d \right),$$

5 $$D1_{\text{D}} \left( d \right) = \left\{ {\begin{array}{*{20}l} 1 \hfill & {if{\kern 1pt} {\kern 1pt} {\kern 1pt} d = D,{\kern 1pt} } \hfill \\ {\max \left\{ {\Delta * D1_{D} \left( {d^{^{\prime}} } \right)\left| {d^{^{\prime}} \in children} \right.of{\kern 1pt} d} \right\}} \hfill & {if{\kern 1pt} {\kern 1pt} {\kern 1pt} d \ne D{\kern 1pt} ,} \hfill \\ \end{array} } \right.$$

where $\Delta$ represents the semantic contribution factor and $D1_{D} \left( d \right)$ is the contribution of disease $d$. For each disease $d$, its contribution to itself is 1, and the contribution of its child node decreases with increasing distance. Obviously, when the two diseases have a larger shared part in their $DAGs$, they will obtain a greater similarity score. $SV\left( {d_{i} } \right)$ and $SV\left( {d_{j} } \right)$ represent the semantic similarity values of $d_{i}$ and $d_{j}$, respectively. Thus, the semantic similarity score of the two diseases $d_{i}$ and $d_{j}$ can be calculated as follows:

6 $$S_{d} \left( {d_{i} ,d_{j} } \right) = \frac{{\sum\nolimits_{{t \in T\left( {d_{i} } \right) \cap T\left( {d_{j} } \right)}} {\left( {D_{{d_{i} }} \left( t \right) + D_{{d_{j} }} \left( t \right)} \right)} }}{{SV\left( {d_{i} } \right) + SV\left( {d_{j} } \right)}}.$$

### Gaussian Interaction Profile Kernel for miRNAs and diseases

**Fig. 5:**
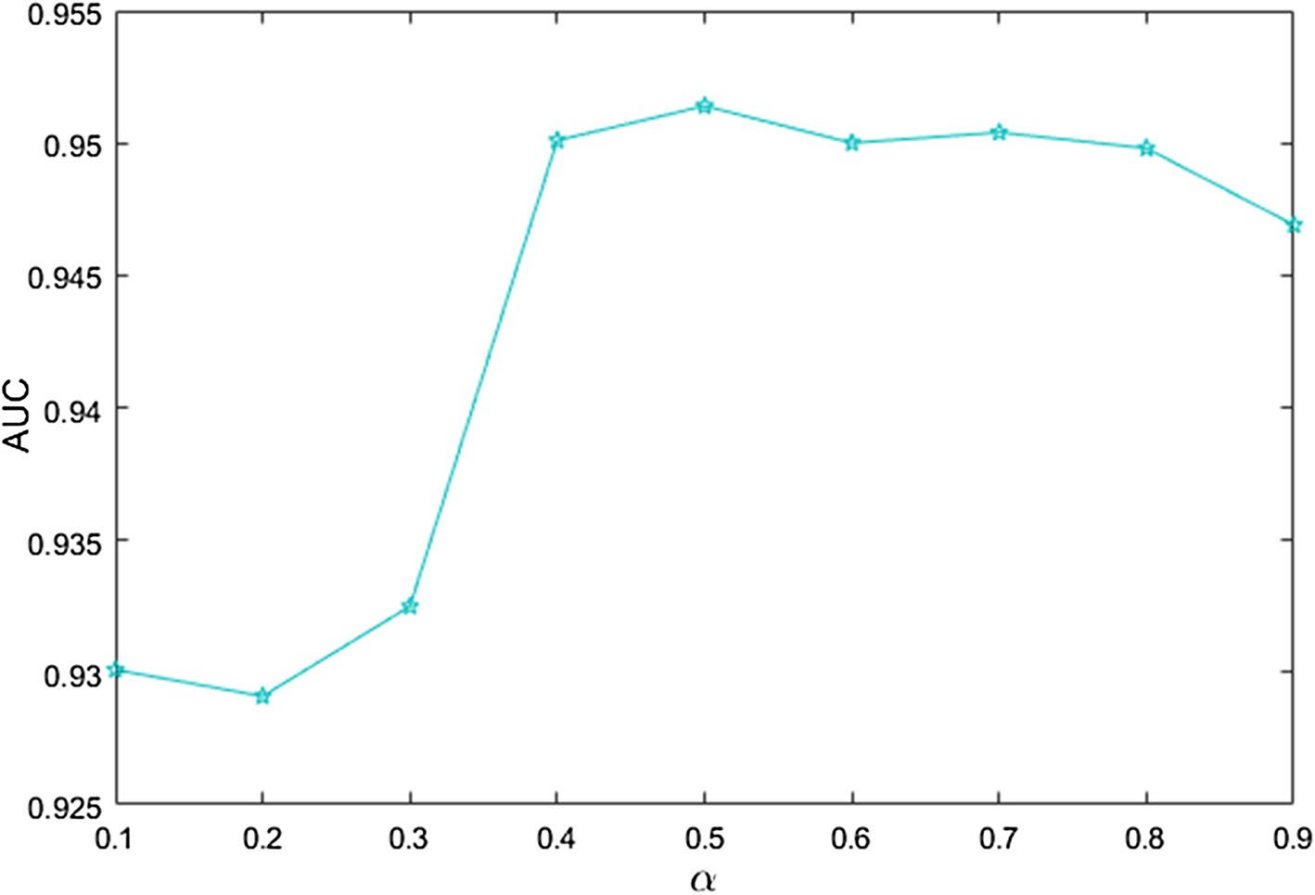
Sensitivity analysis for $\alpha$

According to the previous work [38], the method is based on the idea that it relies on the topological structure of known miRNA–disease associations in a network to compute the similarity of diseases and miRNAs [26]. Here are two miRNAs $m_{i}$ and $m_{j}$ and two diseases $d_{i}$ and $d_{j}$. The network similarity between them can be calculated with the following formulas:

7 $$GIP_{miRNA} \left( {m_{i} ,m_{j} } \right) = \exp \left( { - \gamma \left\| {{\mathbf{Y}}\left( {m_{i} } \right) - {\mathbf{Y}}\left( {m_{j} } \right)} \right\|^{2} } \right),$$

8 $$GIP_{disease} \left( {d_{i} ,d_{j} } \right) = \exp \left( { - \gamma \left\| {{\mathbf{Y}}\left( {d_{i} } \right) - {\mathbf{Y}}\left( {d_{j} } \right)} \right\|^{2} } \right),$$

where $\gamma$ is an adjustable parameter that can control the bandwidth of the kernel. In addition, ${\mathbf{Y}}\left( {m_{i} } \right)$ and ${\mathbf{Y}}\left( {m_{j} } \right)$ are the miRNA interaction profiles of $m_{i}$ and $m_{j}$, respectively. Similarly, ${\mathbf{Y}}\left( {d_{i} } \right)$ and ${\mathbf{Y}}\left( {d_{j} } \right)$ are the disease interaction profiles of $d_{i}$ and $d_{j}$, respectively. Then, the network similarity matrix ${\mathbf{K}}_{m}$ of miRNA and the ${\mathbf{K}}_{d}$ of disease are obtained by combining the original matrix ${\mathbf{S}}_{m}$ and ${\mathbf{S}}_{d}$. The detailed descriptions are as below:

9 $${\mathbf{K}}_{m} = \alpha {\mathbf{S}}_{m} + \left( {1 - \alpha } \right){\mathbf{GIP}}_{miRNA} ,$$

10 $${\mathbf{\rm K}}_{d} = \alpha {\mathbf{S}}_{d} + \left( {1 - \alpha } \right){\mathbf{GIP}}_{disease} ,$$

where $\alpha$ is an adjustable parameter range in [0, 1], and ${\mathbf{K}}_{m}$ represents the miRNA integrated similarity matrix, which is a linear combination of the Gaussian interaction profile kernel similarity for miRNA ${\mathbf{GIP}}_{miRNA}$ and the miRNA functional matrix ${\mathbf{S}}_{m}$. Similar to ${\mathbf{K}}_{m}$, ${\mathbf{K}}_{d}$ represents the disease integrated similarity matrix, which is a linear combination of the Gaussian interaction profile kernel similarity for disease ${\mathbf{GIP}}_{disease}$ and the disease semantic matrix ${\mathbf{S}}_{d}$. When $\alpha$ is equal to 0.5, BGCMF achieves the highest AUC value. The sensitivity analysis of $\alpha$ is shown in Fig. 5.

### Bipartite graph method

Based on the assumption that miRNAs that are similar will interact with similar diseases, the interaction profile for a new miRNA candidate could be inferred from the known interactions of their neighbours. MiRNAs with large similarities to new potential miRNAs are said to be their neighbours. Therefore, we introduce the nearest profile (NP) to our method [39]. Below are the formulas for calculating a new miRNA $m_{i}$ and a new disease $d_{i}$.

11 $${\mathbf{N}}_{m} \left( {m_{i} } \right) = {\mathbf{K}}_{m} \left( {m_{i} ,m_{nearest} } \right) \times {\mathbf{Y}}\left( {m_{nearest} } \right),$$

12 $${\mathbf{N}}_{d} \left( {d_{i} } \right) = {\mathbf{K}}_{m} \left( {d_{i} ,d_{nearest} } \right) \times {\mathbf{Y}}\left( {d_{nearest} } \right),$$

where $m_{nearest}$ and $d_{nearest}$ are the miRNAs most similar to $m_{i}$ and the diseases most similar to $d_{i}$, respectively. ${\mathbf{N}}_{m} \left( {m_{i} } \right)$ and ${\mathbf{N}}_{d} \left( {d_{i} } \right)$ are the association profiles of the miRNAs and diseases, respectively. The NP process in this method can be divided into four steps. First, remove the self-similarity of miRNA matrices ${\mathbf{K}}_{m}$ and ${\mathbf{K}}_{d}$. Next, obtain the nearest neighbour for each miRNA and disease. Then, ignore all miRNA similarities and disease similarities. Finally, the miRNA nearest neighbour matrix ${\mathbf{\rm N}}_{m}$ and disease nearest neighbour matrix ${\mathbf{N}}_{d}$ can be obtained.

### Weighted profile

The weighted profile (WP) is proposed as a simple predictive model in [39]. The idea of the weighted profile is to perform a similarity-weighted average of all other miRNAs or diseases to obtain the prediction matrix. For instance, the WP for a new miRNA $m_{i}$ and a new disease are computed as:

13 $$\widehat{{\mathbf{Y}}}\left( {m_{i} } \right) = \frac{{\sum\nolimits_{j = 1}^{{n_{m} }} {{\mathbf{N}}_{m} \left( {m_{i} ,m_{j} } \right) \times {\mathbf{Y}}\left( {m_{j} } \right)} }}{{\sum\nolimits_{j = 1}^{{n_{m} }} {{\mathbf{N}}_{m} \left( {m_{i} ,m_{j} } \right)} }},$$

14 $$\widehat{{\mathbf{Y}}}\left( {d_{i} } \right) = \frac{{\sum\nolimits_{j = 1}^{{n_{d} }} {{\mathbf{N}}_{d} \left( {d_{i} ,d_{j} } \right) \times {\mathbf{Y}}\left( {d_{j} } \right)} }}{{\sum\nolimits_{j = 1}^{{n_{d} }} {{\mathbf{N}}_{d} \left( {d_{i} ,d_{j} } \right)} }},$$

where ${\mathbf{N}}_{m}$ and ${\mathbf{N}}_{d}$ are the nearest neighbour matrices we construct for miRNA and disease. ${\mathbf{Y}}\left( {m_{j} } \right)$ and ${\mathbf{Y}}\left( {d_{j} } \right)$ are association matrices of miRNA $m_{j}$ and disease $d_{j}$, respectively. First, the BG algorithm is used to obtain the neighbour information about miRNAs and diseases, and then predictions from both miRNA and disease sides are averaged to obtain the final prediction matrix:

15 $${\mathbf{Y}}_{1} = \frac{{\widehat{{\mathbf{Y}}}\left( {m_{i} } \right) + \widehat{{\mathbf{Y}}}\left( {d_{j} } \right)}}{2}.$$

### BGCMF for MiRNA-disease associations association prediction

The traditional collaborative matrix factorization (CMF) method is effective in predicting the underlying interactions between miRNAs and diseases [29]. The objective function of CMF method is defined as:

16 $$\min_{{{\mathbf{A}},{\mathbf{B}}}} = \left\| {{\mathbf{Y}} - {\mathbf{AB}}^{T} } \right\|_{F}^{2} + \lambda_{l} \left( {\left\| {\mathbf{A}} \right\|_{F}^{2} + \left\| {\mathbf{B}} \right\|_{F}^{2} } \right) + \lambda_{d} \left\| {{\mathbf{S}}_{m} - {\mathbf{AA}}^{T} } \right\|_{F}^{2} + \lambda_{t} \left\| {{\mathbf{S}}_{d} - {\mathbf{BB}}^{T} } \right\|_{F}^{2} ,$$

where $\lambda_{l} {\kern 1pt} {\kern 1pt} {\kern 1pt}$, $\lambda_{d} {\kern 1pt} {\kern 1pt}$, and $\lambda_{t}$ are non-parameters and $\left\| \cdot \right\|_{F}^{2}$ represents the Frobenius norm. In this formula, the first item is used to find the low-rank matrices ${\mathbf{A}}$ and ${\mathbf{B}}$ of the reconstructed ${\mathbf{Y}}$. The second item is the Tikhonov regularization term. The last two items are regularization terms that demand potential feature vectors of similar miRNAs/diseases to be similar and potential feature vectors of dissimilar miRNAs/diseases to be dissimilar. However, traditional CMF does not take into account the network relationship between the miRNA and the disease, which will reduce the accuracy of predicting MDAs. Therefore, we introduce the Gaussian kernel similarity ${\mathbf{K}}_{m}$ of miRNA and the ${\mathbf{K}}_{d}$ of disease into CMF [40]. The objective function can be rewritten as:

17 $$\min_{{{\mathbf{A}},{\mathbf{B}}}} = \left\| {{\mathbf{Y}} - {\mathbf{AB}}^{T} } \right\|_{F}^{2} + \lambda_{l} \left( {\left\| {\mathbf{A}} \right\|_{F}^{2} + \left\| {\mathbf{B}} \right\|_{F}^{2} } \right) + \lambda_{d} \left\| {{\mathbf{K}}_{m} - {\mathbf{AA}}^{T} } \right\|_{F}^{2} + \lambda_{t} \left\| {{\mathbf{K}}_{d} - {\mathbf{BB}}^{T} } \right\|_{F}^{2} ,$$

where $\left\| \cdot \right\|_{F}^{2}$ is the Frobenius norm. $\lambda_{l} {\kern 1pt} {\kern 1pt} {\kern 1pt}$, $\lambda_{d} {\kern 1pt} {\kern 1pt}$ and $\lambda_{t}$ represent the positive parameters. In this study, the setting of the three parameters is done by cross-validation. The grid search is adopted to select the optimal parameters among these values:$\lambda_{l} \in \left\{ {2^{ - 2} ,2^{ - 1} ,2^{0} ,2^{1} ,2^{2} } \right\}$, $\lambda_{d} /\lambda_{t} \in \left\{ {2^{ - 6} ,2^{ - 5} ,2^{ - 4} ,2^{ - 3} ,2^{ - 2} ,2^{ - 1} ,2^{0} ,2^{1} ,2^{2} } \right\}$. The association matrix ${\mathbf{Y}}$ is decomposed into two low-rank matrices ${\mathbf{A}}$ and ${\mathbf{B}}$, where ${\mathbf{Y}} \approx {\mathbf{AB}}^{T}$. Tikhonov regularization is adopted to minimize the norms of both ${\mathbf{A}}$ and ${\mathbf{B}}$. The roles of the third and fourth terms are to minimize the squared error ${\mathbf{S}}_{m} \approx {\mathbf{AA}}^{T}$ and ${\mathbf{S}}_{d} \approx {\mathbf{BB}}^{T}$, respectively.

### Initialization of ${\mathbf{A}}$ and ${\mathbf{B}}$

In the CMF method, the first step is to initialize the adjacency matrix ${\mathbf{Y}}$. We use singular value decomposition (SVD) to decompose the input matrix ${\mathbf{Y}} \in {\mathbb{R}}^{n \times m}$ into ${\mathbf{U}}^{n \times k}$, ${\mathbf{S}}^{k \times k}$ and ${\mathbf{V}}^{k \times m}$. Then, matrix ${\mathbf{A}}$ and matrix ${\mathbf{B}}$ are obtained by the following formula:

18 $$\left[ {{\mathbf{U,S,V}}} \right] = SVD\left( {{\mathbf{Y}},k} \right),\quad {\mathbf{A}} = {\mathbf{US}}_{k}^{1/2} ,\quad {\mathbf{B}} = {\mathbf{VS}}_{k}^{1/2} ,$$

where ${\mathbf{S}}$ is a diagonal matrix and ${\text{k}}$ represents the maximum number of singular values.

### Alternating least squares

In this study, alternating least squares is used to optimize ${\mathbf{A}}$ and ${\mathbf{B}}$ until convergence. Here, $L$ is used to represent the objective function of BGCMF. Then, ${\mathbf{A}}$ and ${\mathbf{B}}$ are obtained by letting $\partial L/\partial {\mathbf{A}} = 0,$ and $\partial L/\partial {\mathbf{B}} = 0,$ respectively. Moreover, the optimal values of $\lambda_{l}$, $\lambda_{d}$ and $\lambda_{{\text{t}}}$ are automatically obtained through a fivefold cross-validation experiment. The iterative formulas for ${\mathbf{A}}$ and ${\mathbf{B}}$ are represented by:

19 $${\mathbf{A}} = \left( {{\mathbf{YB}} + \lambda_{d} {\mathbf{K}}_{m} {\mathbf{A}}} \right)\left( {{\mathbf{B}}^{T} {\mathbf{B}} + \lambda_{l} {\mathbf{I}}_{k} + \lambda_{d} {\mathbf{AA}}^{T} } \right)^{ - 1} ,$$

20 $${\mathbf{B}} = \left( {{\mathbf{Y}}^{T} {\mathbf{A}} + \lambda_{{\text{t}}} {\mathbf{K}}_{d} {\mathbf{B}}} \right)\left( {{\mathbf{A}}^{T} {\mathbf{A}} + \lambda_{l} {\mathbf{I}}_{k} + \lambda_{d} {\mathbf{B}}^{T} {\mathbf{B}}} \right)^{ - 1} .$$

Finally, the final prediction matrix ${\mathbf{Y}}$ is obtained by combining both the BG algorithm and the optimized CMF model.

## Data Availability

The datasets that support the findings of this study are available in https://github.com/zhoufeng-coder/. The origins of the data used in the Case Studies in this paper are available on open-source data PMID: 24194601 (http://www.cuilab.cn/hmdd).
